# Supplementary material for: Population structure and microbial community diversity of two common tetillid sponges in a tropical reef lagoon
Source: PeerJ. 2020 Apr 22;8:e9017. doi: 10.7717/peerj.9017 (PMC7183310; doi:10.7717/peerj.9017)
Supplement: Supplemental Information 1 [file peerj-08-9017-s001.docx]

**Supplementary Materials**

**Population structure and microbial community diversity of two common tetillid sponges in a tropical reef lagoon**

Jake Ivan P. Baquiran, Michael Angelou L. Nada, Niño Posadas, Dana P. Manogan, Patrick C. Cabaitan and Cecilia Conaco*

Marine Science Institute, University of the Philippines Diliman, Quezon City, 1101, Philippines

*Corresponding author: Cecilia Conaco (cconaco@msi.upd.edu.ph)

**Supplementary Figures**


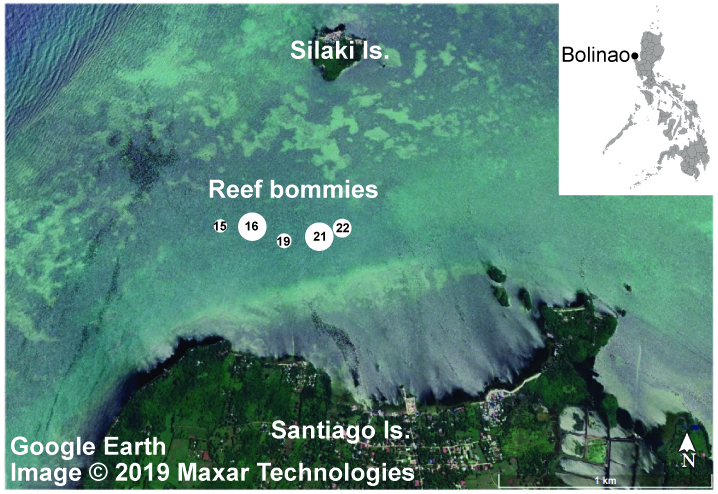


**Supplementary Figure 1**. **Study site.** Tetillid sponge surveys were conducted on five coral bommies (bommie 15, 16, 19, 21, and 22) in the Santiago reef flat in Bolinao, Pangasinan, northwestern Philippines (inset). Sizes of the circles represent the relative diameters of the bommies. Map data from Google, Maxar Technologies © 2019.


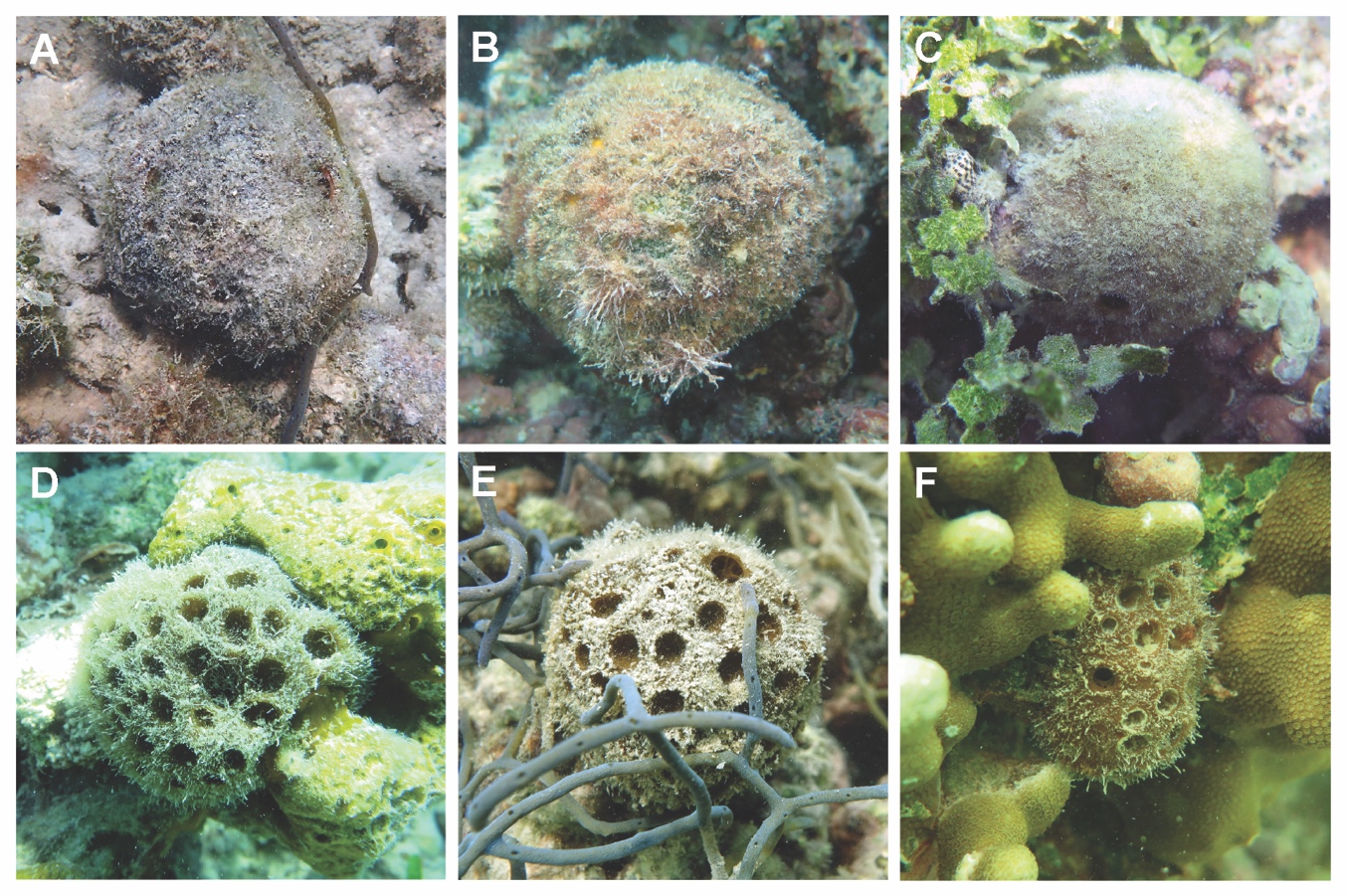


**Supplementary Figure 2.** Tetillid sponges are typically covered by sediments (A) or turf algae (B), and can be found in close interaction with other types of algae (C), sponges (D-E), and corals (F). Individuals in A-C are putative *Cinachyrella* sp. while those in D-F are putative *Paratetilla* sp..

**Supplementary Figure 3.** Phylogenetic tree constructed using CO1 gene sequences. Node support values are posterior probabilities from Bayesian inference analysis using MrBayes. Sequences generated by this study are shown in pink (*Paratetilla* sp.) and blue (*Cinachyrella* sp.) while other sequences were obtained from Szitenberg et al. (2013).

**Supplementary Figure 4.** Rarefaction analysis of *Cinachyrella* sp. and *Paratetilla* sp. 16S rRNA ASVs.

**
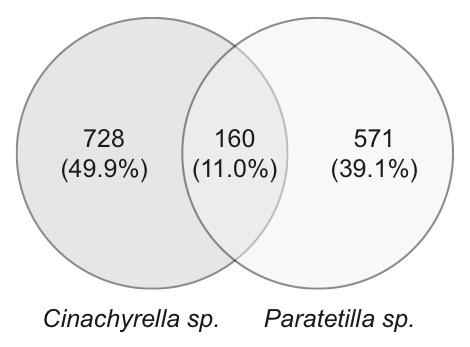
**

**Supplementary Figure 5.** Venn diagram illustrating the number of ASVs shared between *Cinachyrella* sp. and *Paratetilla* sp..

**Supplementary Figure 6.** Phylum (A) and class (B) level microbial community composition of *Cinachyrella* sp*.* and *Paratetilla* sp.. Phyla or classes representing less than 0.4% of the total community are represented as “other microbial phyla or classes.” Colored bars represent the relative abundance of microbial taxa in each replicate sample.

**
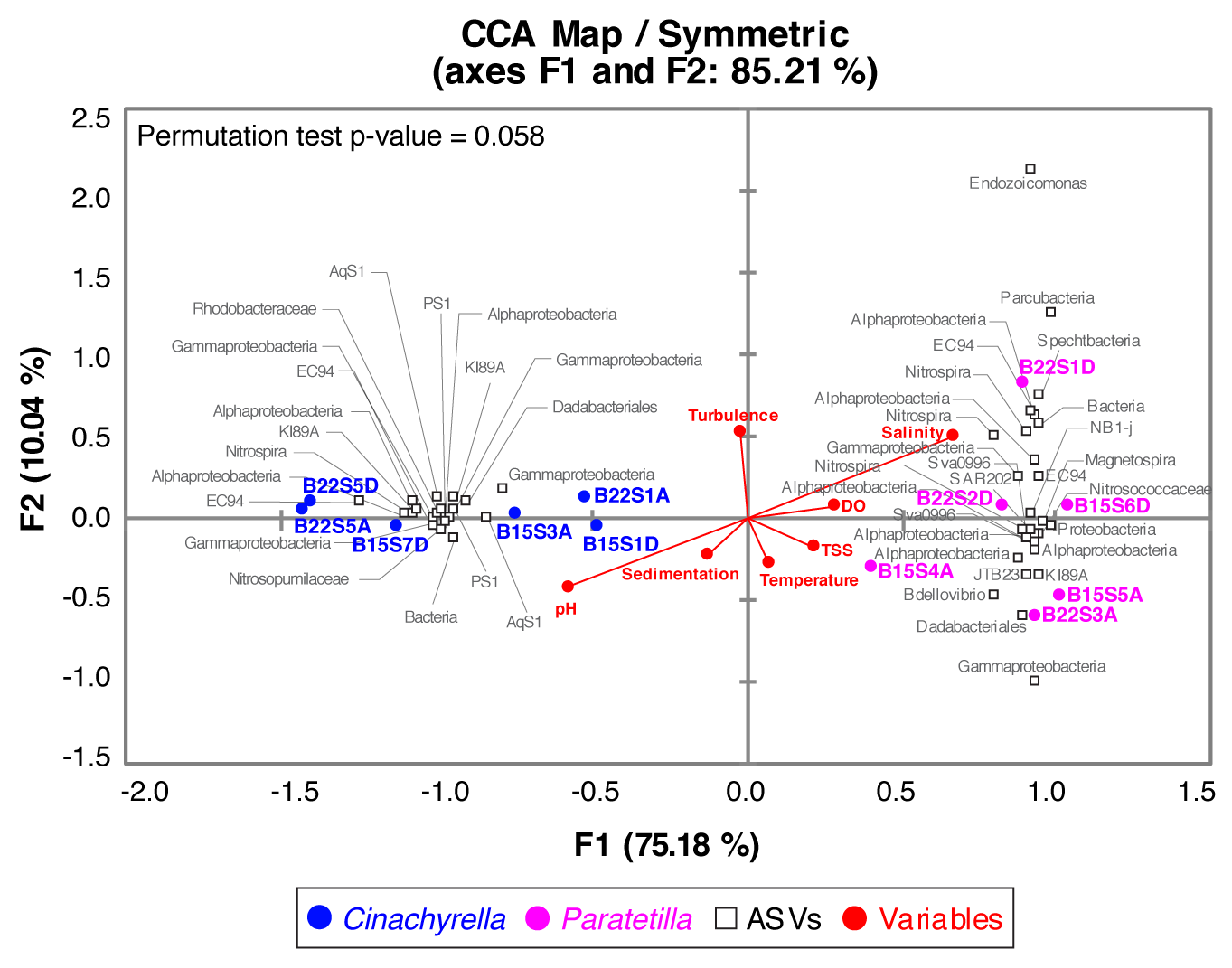
**

**Supplementary Figure 7.** Canonical Correspondence Analysis (CCA) plots showing the relationship between the differentially abundant ASVs (black squares) and measured environmental variables (red circles and lines). Differentially abundant ASVs clustered more closely by sponge species rather than with any environmental parameters. Individual samples are indicated by colored circles (blue, *Cinachyrella* sp.; pink, *Paratetilla* sp.). The CCA plot was generated using XLSTAT version 2020.1.

**Supplementary Tables**

**Supplementary Table 1.** Summary results of the GLM tests for a) tetillid sponge densities between bommies and time, b) tetillid sponge densities between bommies and species, and c) environmental parameters between bommies.

|  |  |  |  | **Factors** |  | **df** | **F** | **p** |
| --- | --- | --- | --- | --- | --- | --- | --- | --- |
| a) Comparison of tetillid sponge density between bommies and time | | | | | | | | |
|  |  |  |  | Bommie |  | 3 | 5.95 | **<0.05** |
|  |  |  |  | Time |  | 2 | 2.39 | 0.09 |
|  |  |  |  | Bommie*Time | | 11 | 0.88 | 0.56 |
|  |  |  |  |  |  |  |  |  |
| b) Comparison of tetillid sponge density between bommies and species | | | | | | | | |
|  |  |  |  | Bommie |  | 4 | 4.75 | **0.001** |
|  |  |  |  | Species |  | 1 | 5.34 | **0.02** |
|  |  |  |  | Bommie*Species | | 4 | 11.60 | **<0.001** |
|  |  |  |  |  |  |  |  |  |
| c) Comparison of different environmental parameters between bommies | | | | | | | | |
|  | pH |  |  | Bommie |  | 4 | 0.22 | 0.92 |
|  | Temperature | |  | Bommie |  | 4 | 0.05 | 0.99 |
|  | Salinity |  |  | Bommie |  | 4 | 0.03 | 1.00 |
|  | Dissolved oxygen | |  | Bommie |  | 4 | 0.09 | 0.99 |
|  | Water turbulence | |  | Bommie |  | 4 | 2.47 | 0.05 |
|  | Sedimentation | |  | Bommie |  | 4 | 1.05 | 0.38 |
|  | Total suspended solids | | | Bommie |  | 4 | 1.19 | 0.32 |
|  |  |  |  |  |  |  |  |  |

**Supplementary Table 2.** Summary results of the K-S tests for pairwise differences in size frequency distribution of tetillid sponges between years per bommie.

| **Bommie** | **Sampling periods** | | | |
| --- | --- | --- | --- | --- |
|  |  | **2017** | **2018** | **2019** |
| Bommie 15 | 2016 | ns | ns | ns |
|  | 2017 |  | ns | ns |
|  | 2018 |  |  | ns |
|  |  |  |  |  |
| Bommie 16 |  | **2017** | **2018** | **2019** |
|  | 2016 | no data | no data | no data |
|  | 2017 |  | **p < 0.001** | ns |
|  | 2018 |  |  | **p < 0.001** |
|  |  |  |  |  |
| Bommie 19 |  | **2017** | **2018** | **2019** |
|  | 2016 | ns | **p < 0.025** | **p < 0.05** |
|  | 2017 |  | **p < 0.05** | **p < 0.05** |
|  | 2018 |  |  | **p < 0.005** |
|  |  |  |  |  |
| Bommie 21 |  | **2017** | **2018** | **2019** |
|  | 2016 | **p < 0.005** | **p < 0.005** | **p < 0.01** |
|  | 2017 |  | **p < 0.001** | ns |
|  | 2018 |  |  | **p < 0.005** |
|  |  |  |  |  |
| Bommie 22 |  | **2017** | **2018** | **2019** |
|  | 2016 | ns | **p < 0.001** | **p < 0.001** |
|  | 2017 |  | **p < 0.001** | **p < 0.001** |
|  | 2018 |  |  | **p < 0.001** |
|  |  |  |  |  |

**Supplementary Table 3.** Environmental parameters at the study site collected quarterly from September 2018 to March 2019.

| **Parameter** | **Bommie** | **Average (±SD)** |
| --- | --- | --- |
| DO (mg/L) | 15 | 4.82 ± 2.46 |
|  | 16 | 4.60 ± 2.26 |
|  | 19 | 4.95 ± 0.86 |
|  | 21 | 5.16 ± 1.11 |
|  | 22 | 5.08 ± 0.92 |
| pH | 15 | 7.59 ± 0.59 |
|  | 16 | 7.79 ± 0.52 |
|  | 19 | 7.59 ± 0.58 |
|  | 21 | 7.78 ± 0.49 |
|  | 22 | 7.82 ± 0.57 |
| Temperature (°C) | 15 | 29.56 ± 2.04 |
|  | 16 | 29.24 ± 1.61 |
|  | 19 | 29.26 ± 1.77 |
|  | 21 | 29.24 ± 1.79 |
|  | 22 | 29.06 ± 1.82 |
| Salinity (ppt) | 15 | 30.48 ± 1.10 |
|  | 16 | 30.24 ± 1.62 |
|  | 19 | 30.50 ± 1.50 |
|  | 21 | 30.56 ± 1.63 |
|  | 22 | 30.58 ± 2.23 |
| Clod card (% weight loss) | 15 | 0.16 ± 0.02 |
|  | 16 | 0.19 ± 0.10 |
|  | 19 | 0.15 ± 0.03 |
|  | 21 | 0.16 ± 0.03 |
|  | 22 | 0.17 ± 0.04 |
| Sedimentation rate (g/m2/d) | 15 | 32.49 ± 15.24 |
|  | 16 | 36.66 ± 27.67 |
|  | 19 | 31.57 ± 17.17 |
|  | 21 | 35.91 ± 11.31 |
|  | 22 | 29.07 ± 9.40 |
| Total suspended solids (g/L) | 15 | 0.04 ± 0.02 |
|  | 16 | 0.06 ± 0.09 |
|  | 19 | 0.04 ± 0.03 |
|  | 21 | 0.06 ± 0.11 |
|  | 22 | 0.03 ± 0.02 |

**Supplementary Table 4.** Morphological characteristics of the tetillid sponges.

| **Criteria** | **Tetillid species 1** | **Tetillid species 2** |
| --- | --- | --- |
| Color of the tissue (cross section) | Yellow | Brown |
| Porocalices (floor characteristic) | Usually with oscular tubes | No oscular tubes observed |
| Porocalices (shape) | Hemi-spherical | Hemi-spherical or narrow; sometimes closed |
| Oxea | Usually present | Usually present |
| Anatriene | Usually present | Usually present |
| Triradiate symmetrical rays | Usually absent | Usually present |
| Protriane | Usually present | Present |
| C-sigma | Usually present | Usually present |
| S-sigma | Usually present | Usually present |
| Microxea | Usually present | Usually present |
| ID | *Cinachyrella* sp. | *Paratetilla* sp. |

**Supplementary Table 5.** PERMANOVA (using Adonis method) and ANOSIM results for the comparison of microbial communities at ASV level between the two species, samples of each species collected at different times, and samples of each species collected from different bommies. Significant p-values are shown in bold font.

|  | PERMANOVA | | ANOSIM | |
| --- | --- | --- | --- | --- |
|  | *R^2^* | p-value | *R* | p-value |
| Species  (*Cinachyrella* sp.: *Paratetilla* sp.) | 0.84118 | **0.001** | 0.874 | **0.001** |
| Time *(Cinachyrella* sp.*)*  (December 2016 : April 2017) | 0.35515 | 0.1 | 0.4815 | 0.1 |
| Time *(Paratetilla* sp.*)*  (December 2016 : April 2017) | 0.51471 | 0.1 | 0.8148 | 0.1 |
| Bommie *(Cinachyrella* sp.*)*  (Bommie 15: Bommie 22) | 0.28559 | 0.3 | 0.2593 | 0.3 |
| Bommie (*Paratetilla* sp.*)*  (Bommie 15: Bommie 22) | 0.287 | 0.4 | 0.1852 | 0.4 |

**Supplementary Table 6.** PERMANOVA (using Adonis method) and ANOSIM results for the comparison of the predicted KO pathways in the two sponge species and between samples of each species collected at different times.

|  | PERMANOVA | | ANOSIM | |
| --- | --- | --- | --- | --- |
|  | *R^2^* | p-value | *R* | p-value |
| Species  (*Cinachyrella* sp. : *Paratetilla* sp.) | 0.79417 | **0.003** | 1 | **0.002** |
| Time *(Cinachyrella* sp.*)*  (December 2016 : April 2017) | 0.11446 | 0.8 | 0.2593 | 0.9 |
| Time *(Paratetilla* sp.*)*  (December 2016 : April 2017) | 0.48786 | 0.1 | 0.5926 | 0.1 |
